# Complete mitochondrial genome of the double-lined fusileer, *Pterocaesio digramma* (Perciformes, Caesionidae): mitogenome characterization and phylogenetic analysis

**DOI:** 10.1080/23802359.2020.1778575

**Published:** 2020-07-02

**Authors:** Ha Yeun Song, Yun-Hwan Jung, Bora Kim, Young Ji Choi, Tu Van Nguyen, Dae-Sung Lee

**Affiliations:** a Department of Genetic Resources Research, National Marine Biodiversity Institute of Korea, Seocheon-Gun, Republic of Korea; b International Center for Marine Biodiversity, National Marine Biodiversity Institute of Korea, Seocheon-Gun, Republic of Korea; c Department of Ecology, Institute of Tropical Biology, Vietnam Academy of Science and Technology, Ho Chi Minh City, Vietnam

**Keywords:** Mitochondrial genome, Perciformes, Caesionidae, *Pterocaesio digramma*

## Abstract

The complete mitochondrial genome of the double-lined fusileer, *Pterocaesio digramma*, which belongs to the family Caesionidae was determined. The complete mitochondrial genome has a length of 16,504 bp and consists of 13 protein-coding genes, 22 tRNA genes, 2 rRNA genes, and a control region. *P. digramma* has a mitochondrial gene arrangement that is typical of vertebrates. Phylogenetic analysis using mitochondrial genomes of 15 related species revealed that *P. digramma* formed a well-supported monophyletic group with the other Caesionidae and Lutjanidae species.

The double-lined fusileer, *Pterocaesio digramma* (Perciformes, Caesionidae), is a tropical reef-associated marine fish that inhabits the Western Pacific, ranging from Indonesia to Western Australia, New Caledonia, and northwards to Japan. This species is listed as Least Concern in IUCN Red List due to overfishing and harvesting aquatic resources (Russell et al. 2016). Although previous studies reported Caesionidae members was nested within the Lutjanidae (Johnson 1993; Miller and Cribbs 2007; Guo et al. 2014), its taxonomic position within Lutjanidae is not clear. In the present study, we determined the complete mitochondrial DNA sequence of *P. digramma* and analyzed the phylogenetic relationship of this species with members of Caesionidae and Lutjanidae.

The *P. digramma* specimen was collected from Ho Chi Minh City, Vietnam (10.53 N, 106.45 W). Total genomic DNA was extracted from the specimen tissue, which has been deposited at the National Marine Biodiversity Institute of Korea (Voucher No. MABIK0002407). The mitogenome was sequenced using Illumina Hiseq 4000 sequencing platform (Illumina, San Diego, CA, USA) and assembled with *SOAPdenovo* at Macrogen Inc. (Korea). The complete mitochondrial genome was annotated using MacClade ver. 4.08 (http://macclade.org/macclade; Maddison and Maddison 2005) and tRNAscan-SE ver. 2.0 (http://lowelab.ucsc.edu/tRNAscan-SE; Lowe and Chan 2016).

The complete mitochondrial genome of *P. digramma* (GenBank accession no. LC549803) is 16,504 bp in length and includes 13 protein-coding genes, 22 tRNA genes, 2 rRNA genes, and a control region. The overall base composition is 27.87% A, 30.87% C, 16.50% G, and 24.76% T. Similar to the mitogenomes of other vertebrates, the AT content is higher than the GC content (Saccone et al. 1999). All tRNA genes can fold into a typical cloverleaf structure, with lengths ranging from 67 to 75 bp. The *12S rRNA* (949 bp) and *16S rRNA* genes (1703 bp) are located between tRNA^Phe^ and tRNA^Val^ and between tRNA^Val^ and tRNA^Leu(UUR)^, respectively. Of the 13 protein-coding genes, 12 start with ATG; the exception being *COI*, which starts with GTG. The stop codon of the protein-coding genes is TAA (*COI*, *ATP8*, *ND4L*, and *ND5*), T (*COII*, *ND3*, *ND4*, and *Cytb*), TA (*ND2*, *ATP6*, and *COIII*), and TAG (*ND1* and *ND6*). A control region (825 bp) is located between tRNA^Pro^ and tRNA^Phe^.

The phylogenetic trees were constructed by the maximum-likelihood method using MEGA 7.0 software (MEGA, Philadelphia, PA, USA; Kumar et al. 2016). We compared the phylogenetic trees of the newly sequenced genome and 17 others complete Caesionidae and Lutjanidae species mitochondrial genome sequences acquired from the National Center for Biotechnology Information. We confirmed that *P. digramma* formed a monophyletic group with the other Caesionidae species and the Caesionidae was nested within the Lutjanidae (Figure 1). This mitochondrial genome provides a valuable resource for addressing taxonomic issues and developing conservation strategy.

**Figure 1.**
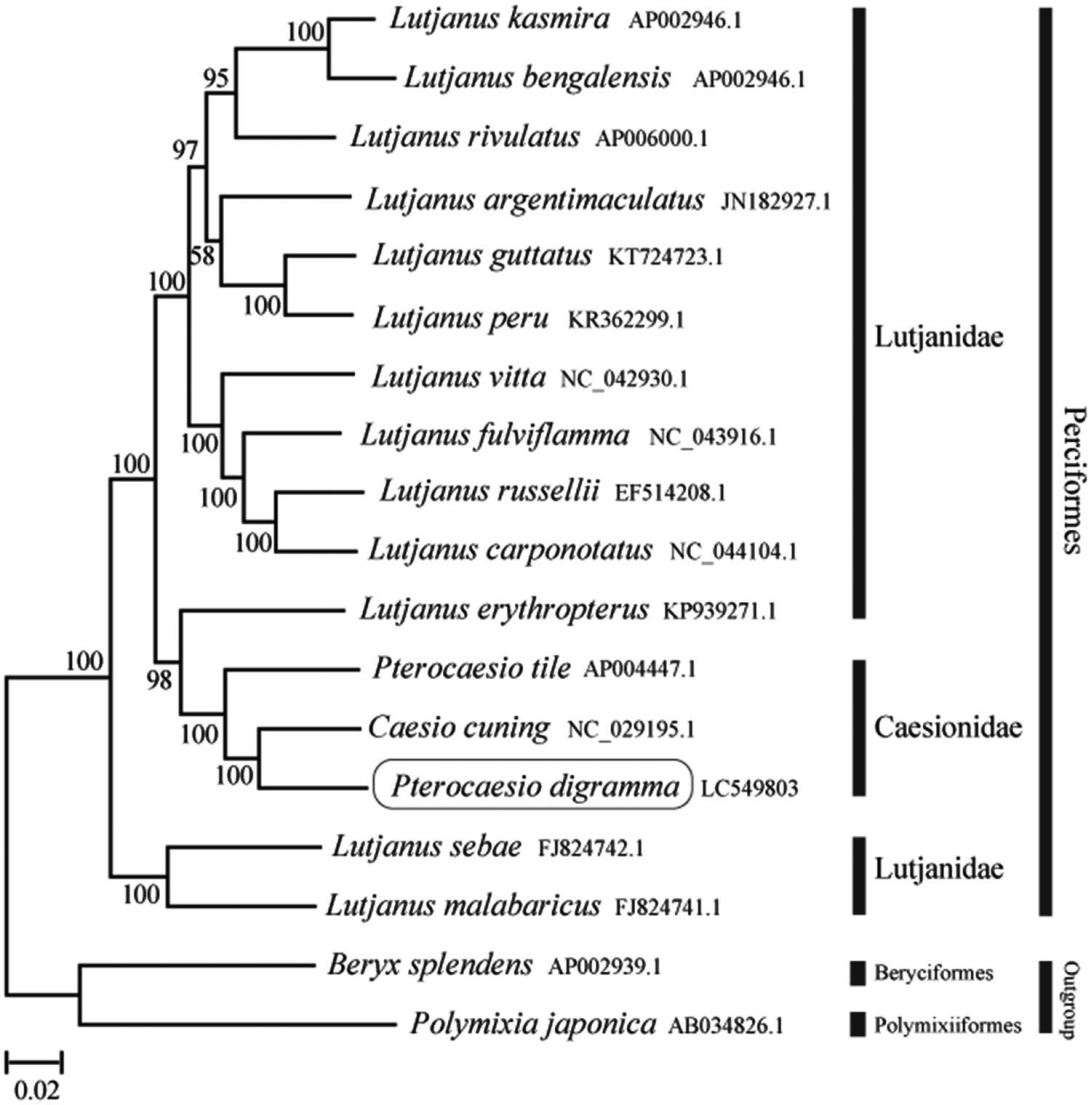
Phylogenetic position of *Pterocaesio digramma* based on a comparison with the complete mitochondrial genome sequences of 15 related species. The analysis was performed using MEGA 7.0 software. The accession number for each species is indicated after the scientific name.

## Data Availability

The data that support the findings of this study are openly available in the DNA Data Bank of Japan (accession no. LC549803) at https://www.ddbj.nig.ac.jp.
